# Supplementary figures and images for: Application of Health Belief Model for the assessment of COVID-19 preventive behavior and its determinants among students: A structural equation modeling analysis
Source: PLoS One. 2022 Mar 21;17(3):e0263568. doi: 10.1371/journal.pone.0263568 (PMC8936445; doi:10.1371/journal.pone.0263568)

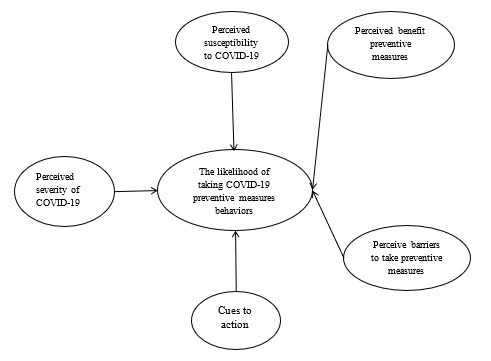

Supplement: S1 Fig — (TIF) [file pone.0263568.s001.tif]
