# Supplementary material for: Application of Health Belief Model for the assessment of COVID-19 preventive behavior and its determinants among students: A structural equation modeling analysis
Source: PLoS One. 2022 Mar 21;17(3):e0263568. doi: 10.1371/journal.pone.0263568 (PMC8936445; doi:10.1371/journal.pone.0263568)
Supplement: Attachment — Submitted filename: Remarks by Reviewer.pdf [file pone.0263568.s002.pdf]

Remarks by Reviewer.

**Criteria**	**Remarks**
1. The study presents the results of original research.	The strongest contribution of this study is the findings. This is because the results show that students in the Ethiopian city of Gondar do not have good practice of Covid-19 preventive measures.
2. Results reported have not been published elsewhere.	As per claim by author/s.
3. Experiments, statistics, and other analyses are performed to a high technical standard and are described in sufficient detail.	Methodology is sound and well executed.<br><br>***However, Post-hoc power analysis should be conducted to ascertain statistical power for sample size adequacy (Refer to lines 123 – 130).***
4. Conclusions are presented in an appropriate fashion and are supported by the data.	Conclusion is logically derived from the analysis of data.<br><br>***However, this reviewer recommends the author/s to improve on the practical recommendations based on the findings of the study. Some thought should be given to what measures should be in place to improve the adoption of good practices of Covid-19 preventive measures among students.***
5. The article is presented in an intelligible fashion and is written in standard English.	This is the weakest part of the article. Numerous typos exist in the manuscript. For example, expression like “pandemic that *eats* many lives (line 66).<br><br>***This article requires editorial work to improve the written quality. Furthermore, the referencing style needs to conform to the standard expected by the Journal.***
6. The research meets all applicable standards for the ethics of experimentation and research integrity.	As claimed by author/s.
7. The article adheres to appropriate reporting guidelines and community standards for data availability.	As claimed by author/s.

Conclusion: ***This reviewer recommends the article to be accepted with minor revision.***
