# Supplementary material for: Application of Health Belief Model for the assessment of COVID-19 preventive behavior and its determinants among students: A structural equation modeling analysis
Source: PLoS One. 2022 Mar 21;17(3):e0263568. doi: 10.1371/journal.pone.0263568 (PMC8936445; doi:10.1371/journal.pone.0263568)
Supplement: Attachment — Submitted filename: Response to editor and reviewers OHBI PLOS ONE.docx [file pone.0263568.s003.docx]

Dear Ali B. Mahmoud, Ph.D., editor and reviewers:

Hereby, we resubmit the enclosed –revised- manuscript ID PONE-D-21-05732-[ EMID:15744fc9849b3e3d], which is entitled “Application of Health Belief Model for the Assessment of COVID-19 Preventive Behavior and its Determinants among Students: A Structural Equation Modeling Analysis” to your journal*.*

We have read the comments carefully and we were able to implement all of them (see the manuscript with track changes). While reading the manuscript critically, we spotted English grammar errors, which we and a language expert have corrected too. We hope that our revision will be felt like an improvement. We certainly feel this manuscript has improved thanks to the suggestions of the reviewers. The datasets generated and/or analyzed in the current study are available publicly with no restriction at <https://www.kaggle.com/kegnieshitu/determinants-of-covid19-preventive-behaviors>

**Response to the Editor’s Comment and suggestions**

On receipt and processing, your manuscript was sent to two reviewers for assessment. I am now in receipt of the recommendation from the two reviewers, which you will find at the bottom of this email. I have also now had the opportunity to re-read the manuscript myself, and I have two additional observations which I wish to offer in conjunction with the reviewers’ reports.

Your work ignores a substantial research body where COVID-19/Pandemic/wartime perceptions were conceptualised, and this work needs to be consulted with. See Mahmoud et al. [1] and Mahmoud and Reisel [2]

Please, whilst revising, offer your manuscript proper proofreading.

References

1. Mahmoud AB, Grigoriou N, Fuxman L, Reisel WD, Hack-Polay D, Mohr I: A generational study of employees’ customer orientation: a motivational viewpoint in pandemic time. Journal of Strategic Marketing 2020:1-18.

2. Mahmoud AB, Reisel WD: Exploring Personal Experience of Wartime Crisis Effects on Job Insecurity in Syria. Psihologia Resurselor Umane 2015, 13(2):245–256.

***Response:*** *Thank you very much, Dr. Ali B. Mahmoud, for your input and comments. I looked at two of the documents and found them both interesting. I tried to incorporate the findings of the first article in the background section of our manuscript* ***(See page 5, line 93-97 in the manuscript****). However, the second article is not related to the present study. While its primary focus is not related to COVID-19, the paper attempted to provide an overview of how job insecurity was in the Syrian war crisis. On the other hand, the present study attempted to provide an overview of how students are implementing the recommended preventive actions for COVID-19. Because of this, we could not consult the second article.*

**Response to Journal requirements:**

When submitting your revision, we need you to address these additional requirements.

Please ensure that your manuscript meets PLOS ONE's style requirements, including those for file naming. The PLOS ONE style templates can be found athttps://journals.plos.org/plosone/s/file?id=wjVg/PLOSOne_formatting_sample_main_body.pdf andhttps://journals.plos.org/plosone/s/file?id=ba62/PLOSOne_formatting_sample_title_authors_affiliations.pdf

***Response:*** *We have prepared the manuscript in accordance with the guidelines presented at the aforementioned web addresses..*

Please provide additional details regarding participant consent. In the ethics statement in the Methods and online submission information, please ensure that you have specified whether consent was informed.

***Response:*** *We referred to the ethics statement and stated that we have obtained ethical approval from the University of Gondar. As well, we obtained consent from each participant >=18 years of age and participants <18 years of age from their parents/guardians and their assent (****See page 14, line 292-300 in the manuscript****).*

Please include your actual numerical p-values in Table 4.

***Response:*** *P-values are included for each correlation coefficient (****See table 4 on page 19 in manuscript****)*

Please revise your tables to replace p-values of "0.000" to "<0.001".

***Response:*** *Correction was made accordingly (****see Table 7 on page 23 in the manuscript****)*

Please provide the names of the five schools were participants were recruited from.

***Response:*** *We described the list of selected schools (****See line 169-171 on page 8 in the manuscript****)*

Please include additional information regarding the survey or questionnaire used in the study and ensure that you have provided sufficient details that others could replicate the analyses. For instance, if you developed the survey or questionnaire as part of this study and it is not under a copyright more restrictive than CC-BY, please include a copy, in both the original language and English, as Supporting Information. If the questionnaire is published, please provide a citation to the (1) questionnaire and/or (2) original publication associated with the questionnaire.

***Response:*** *The tool was prepared by adapting instruments from various studies and that we provided an appropriate citation to the studies associated with the questionnaire. (****See line 197-198 on page 10 in the manuscript****)*

7. We note that you have indicated that data from this study are available upon request. PLOS only allows data to be available upon request if there are legal or ethical restrictions on sharing data publicly. For information on unacceptable data access restrictions, please see http://journals.plos.org/plosone/s/data-availability#loc-unacceptable-data-access-restrictions.

**Response:** We made our dataset publicly available at:

<https://www.kaggle.com/kegnieshitu/determinants-of-covid19-preventive-behaviors>

8. We note you have included a table to which you do not refer in the text of your manuscript. Please ensure that you refer to Table 1 in your text; if accepted, production will need this reference to link the reader to the Table.

***Response:*** *Sorry for the mistake. We cited the table in its appropriate place (****See line 304 on page 14 in the manuscript)***

**Response to reviewer 1 comments and suggestions**

The strongest contribution of this study is the findings. This is because the results show that students in the Ethiopian city of Gondar do not have good practice of Covid-19 preventive measures. Methodology is sound and well executed. However, post-hoc power analysis should be conducted to ascertain statistical power for sample size adequacy (Refer to lines 123 – 130). The conclusion is logically derived from the analysis of data. However, this reviewer recommends the author/s to improve on the practical recommendations based on the findings of the study. Some thought should be given to what measures should be in place to improve the adoption of good practices of Covid-19 preventive measures among students. This is the weakest part of the article. Some typos (in the Table as well) exist in the manuscript. For example, expression like “pandemic that eats many lives" (line 66). This article requires editorial work to improve the written quality. Furthermore, the referencing style needs to conform to the standard expected by the Journal.

***Response:*** *Thank you for your comment. We did a post hoc power analysis based on assumptions including, the amount of examining variable in the endogenous variable (COVID-19 preventive behavior=0.43), the number of predictors (exogenous variables=5), significant level=0.05, and sample size=370. The computation was done using an online statistical calculator which was developed by an American professor, Daniel Soper (1) (****See line 160-164 on page 8 in the manuscript****).*

1. *Soper, D.S. (2021). Post-hoc Statistical Power Calculator for Multiple Regression [Software]. Available from* [*https://www.danielsoper.com/statcalc*](https://www.danielsoper.com/statcalc)

*Moreover, we gave this manuscript to an English language expert, Mr. Fikadie, for an English language edition****.*** *Then* *the entire manuscript was edited for grammatical and typographic errors based on his suggestion and comments. We checked our referencing style whether it is per the journal requirement and made corrections. In addition to this, we tried to correct contextual errors we had noted in our proofreading and emotional expressions like “*pandemic that eats many lives” *were revised (****see line 75-76 on page 4 in the manuscript****).*

**Response to reviewer 2 comments and suggestions**

Reviewer #2: The effort invested in the crafting of this paper is apparent. However, the paper in its current form needs the suggested improvements to overcome the listed limitations and produce a stronger contribution to the state of existing knowledge within a reasonable timeframe. As a result, I am recommending a resubmit addressing the recommended changes for this paper prior to its acceptance in journal. I hope that the review comments will help further improve the work, or reframe it in such a way that a revised submission would be strengthened.

(1) In the abstract and introduction section, the rationale for and the aim/motivation of the study has not been stated, the rationale (academic underpinning) for conducting this study is not strong and is currently weak. E.g. What is the originality of this study?

***Response:*** *Thank you for your valuable suggestions and comments. We tried to further describe the rationale and its originality in the introduction sections, (****see line 113-132 on page 6 in the manuscript****)*

(2) The paper provides a satisfactory literature review demonstrating an understanding of the relevant literature in the field with the appropriate up-to-date literature sources.

***Response:*** *Thank you for your feedback.*

(3) Although the research methodology applied for the study is appropriate, this section lacks academic justifications to support your methodology choices – some of the statements/claims made sounds more arbitrary. There is a need to discuss the alternatives and justification of methods selected for data collection and data analysis, supported by appropriate references need to be provided.

***Response:*** *We discussed the justifications for the data collection and analysis methodology we had used in this study. For instance, we followed a self-administered approach to collect our data for the following reasons; a) all of our participants were literates, b) the self-administered technique is better in reducing social desirability bias over the interviewer-administered approach, and c) the self-administered technique saves time and resource (****see in line 185-188 at page 9 in the manuscript****)*

*Regarding the analysis technique, we employed a structural equation modeling analysis which has been widely used by health behavior researchers investigating complex relationships between latent constructs such as perceived susceptibility, COVID-19 preventive behaviors, and so on. There are two approaches of structural equation modeling approaches; covariance-based SEM (CB-SEM) and variance-based same the so-called partial least squares SEM(PLS-SEM). Even though these approaches have the same purpose, they differ in their basic assumptions. The former is based on the maximum likelihood estimation technique that requires data that meets the multivariate normality assumption. Whereas the latter computes estimates using ordinary least squares estimation techniques regardless normality of the data. Moreover, the PLS-SEM is effective over CB-SEM with a small sample size and/or data that violate multivariate assumptions. In our case, we preferred to use PLS-SEM because our data didn’t meet the multivariate normality assumption (the details of comparison of these methods and supporting articles are presented in the manuscript,* ***see in line 245-363 on page 12****).*

(4) An interesting area of research and the results provide significant theoretical and practical contributions but the discussions section could better relate the findings to entries information management applications.

***Response:*** *We made revisions to the discussion part by further discussing the impact of the findings on actual practice. (****see the discussion part in the revised manuscript)***

(5) The current conclusion section needs to answer the following questions:

• How does the paper bridge the gap between theory and practice?

• What is the impact upon society?

***Response:*** *We tried to mention how the current study findings could brig the gap b/n the theory and practice and its implication of on the society (****See the conclusion part in the revised manuscript and see line 455-471 from page 26-27 in the manuscript****)*

(6) Overall, the paper is well-written and structured, which does much for its readability and comprehensibility.

***Response:*** *Thank you so much!*
