# Supplementary material for: Application of Health Belief Model for the assessment of COVID-19 preventive behavior and its determinants among students: A structural equation modeling analysis
Source: PLoS One. 2022 Mar 21;17(3):e0263568. doi: 10.1371/journal.pone.0263568 (PMC8936445; doi:10.1371/journal.pone.0263568)
Supplement: Attachment — Submitted filename: Response to editor and reviewers PLOS ONE.docx [file pone.0263568.s004.docx]

**Dear Ali B. Mahmoud, Ph.D., editor and reviewers:**

Hereby, we resubmit the enclosed –revised- manuscript ID PONE-D-21-05732-[ EMID:15744fc9849b3e3d], which is entitled “Application of Health Belief Model for the Assessment of COVID-19 Preventive Behavior and its Determinants among Students: A Structural Equation Modeling Analysis” to your journal*.*

We have read the comments carefully and we were able to implement all of them (**see the manuscript with track changes**). While reading the manuscript critically, we spotted English grammar errors, which we and a language expert have corrected too. We hope that our revision will be felt like an improvement. We certainly feel this manuscript has improved thanks to the suggestions of the reviewers.

**Response to the Editor’s Comment and suggestions**

Outstanding from the previous review: AB Mahmoud and WD Reisel [1] conceptualise wartime perceptions which can form the theoretical premise for COVID-19 perceptions since the latter has triggered a war-like context— therefore, it has to be cited. While revising and addressing Reviewer 1 comments, make sure an English native speaker proofreads the whole text.

References

1.            Mahmoud AB, Reisel WD: **Exploring Personal Experience of Wartime Crisis Effects on Job Insecurity in Syria**. *Psihologia Resurselor Umane* 2015, **13**(2):245–256.

***Response:*** *Thank you very much, Dr. Ali B. Mahmoud, for your input and comments. Initially, I was thought that the second manuscript is not related to ours. Now, I realized that the article is contextually related to our study under the umbrella of disaster (COVID-19 vs Wartime). I incorporated the article into my manuscript. (****See line 94-98 in the manuscript****). In addition to this, the whole manuscript was given to English experts in the University for proofreading, and amendments were made based on their feedback (****see the revised manuscript with track changes****)*

**Response to reviewer 1 comments and suggestions**

In my first review of this article, I made comments on the need for post-hoc power analysis. This was included. Furthermore, I commended the authors of the important contribution of the article related to children and Covid-19 prevention behaviors and recommended authors to add relevant practical recommendations. What is unclear is the phrase "behavior change barograms" (line 488). Lastly, this revised submission still requires professional proofreading and editing to improve English expressions and grammar.

***Response:*** *Thank you Dr. Seong-Yuen Toh for your valuable comments and suggestions. Sorry for the error. We said "behavior change barograms" just to say "behavior change programs", it was a typographical error. We made corrections accordingly (****See line 461 in the manuscript****). In addition to this proofreading of the whole manuscript was done by the research team and English language experts. Moreover, our study findings claimed that the COVID-19 preventive practice among students was quite low. Based on these findings, we recommended schools design and implement behavior change interventions to enhance their pupil’s COVID-19 prevention practice. We also suggest some important focus of intervention based on our finding including reduction of barriers through the provision of facemasks, hand sanitizers, and soaps for those who are in financial hardship and enhancing students' self-efficacy to improve their prevention practice (****see the conclusion and recommendation section of the manuscript****).*

**Response to reviewer 2 comments and suggestions**

I am pleased to see that that the authors have taken the previous review comments on board and have set out to deal with every one of them. There has clearly been a great deal of additional work undertaken to address each of the reviewers' concerns, which is duly noted and appreciated. This has made a significant impact on the quality of the paper. Thank you

***Response:*** *Thank you very much for your valuable contribution to the improvement of the manuscript.*
