# Supplementary material for: Application of Health Belief Model for the assessment of COVID-19 preventive behavior and its determinants among students: A structural equation modeling analysis
Source: PLoS One. 2022 Mar 21;17(3):e0263568. doi: 10.1371/journal.pone.0263568 (PMC8936445; doi:10.1371/journal.pone.0263568)
Supplement: Attachment — Submitted filename: Review Plos One Ethiopia Study.docx [file pone.0263568.s005.docx]

1) Suggestion to improve paper’s structure:

I suggest removing the first paragraph that introduces Covid-19. I find it to be too medically focused. There is no need to speculate on the origins of the virus (particularly since it is not established definitively) since it is irrelevant for the purpose of this paper. Alternatively, authors could replace medical wording with simple reference to Covid-19 pandemic. Everyone knows and understands what this is.

# I would suggest instead to incorporate some of the already established early research studies on negative effects of Covid-19 as applicable to either the same settings (such as educational environment), or the same demographics (such as kids). For example, the following paper summarizes the up-to-date research finding based on kids at-home eating habits during Covid:

# “The Janus-faced effects of COVID-19 perceptions on family healthy eating behavior: Parent’s negative experience as a mediator and gender as a moderator” by [Ali B. Mahmoud](https://onlinelibrary.wiley.com/action/doSearch?ContribAuthorStored=Mahmoud%2C+Ali+B), [Dieu Hack-Polay](https://onlinelibrary.wiley.com/action/doSearch?ContribAuthorStored=Hack-Polay%2C+Dieu), [Leonora Fuxman](https://onlinelibrary.wiley.com/action/doSearch?ContribAuthorStored=Fuxman%2C+Leonora), [Maria Nicoletti](https://onlinelibrary.wiley.com/action/doSearch?ContribAuthorStored=Nicoletti%2C+Maria) <https://onlinelibrary.wiley.com/doi/10.1111/sjop.12742>

Giving the reader the summary of findings about Covid related impacts within similar context would present a better introductory background to the current study.

2) I suggest deleting the entire section “Study Period and Setting”. The only immediately relevant information in this section are the dates of the study, which authors can easily incorporate into the next section.

3) Here are some suggestions to correct language throughout the manuscript

Line 73: remove end of sentence punctuation.

Lines 75-78 (third paragraph) : I don’t see the need to re-state mortality statistics as those do change rather quickly and are widely accessible online. This paper is about students in University setting and as such the focus of the introduction should be on the specifics of the environment under consideration.

Line 88-89: grammar correction : “more than 94% of students were following the recommended preventive health behaviors in Iran

Line 106: correct punctuation – remove “; ”

Line 116: grammar correction – “The HBM is one of the various health behavior models that has been…”

Lie 127: replace “study area” with academia.

Line 136: replace “administration” with administered.

Line 147: replace “refers” with refer.

Line 151: and/or

Line 156: capitalize the title of the journal

Line 162: replace punctuation “;” with :

Line 163: coma needed ” … variable (0.43),”

Line 167: “governmental schools, which resulted…”

Line 188: replace punctuation “;” with :

Line 190: insert “is resource efficient”

Line 235: delete coma - “practice concerning handwashing, …”

Line 244: add coma – “cleaning, coding…”

Line 262: insert “ produce precise estimate with smaller…”

Line 276: add coma – “perceived benefit, perceived barrier…”

Lin 301: add reference for Declaration of Helsinki.

Line 312: replace “kept” with keep for grammatical consistency.

Line 324: correct - “…scores were higher than the neutral score…”

Line 337: add coma and lower case – “perceived severity (r = 0.2, p < 0.05), perceived benefit…”

All statistical techniques, new and old, should have reference. For example, line 336 and 365

Line 407-408: poor choice of wording “These results are results lower than the result reported by various previous studies…” Please rewrite this sentence

Line 420: lower case “perceived barriers”

Line 437: “…studies that were done using the health belief model…”

Line 440: use abbreviation HBM instead of health belief model.

Line 446: remove “is” or add our results “… the results are consistent…”

Line 449: replace punctuation “;” with :

Line 457: capitalize the name of the model to keep consistent “Health Belief Model”
