# Supplementary material for: Application of Health Belief Model for the assessment of COVID-19 preventive behavior and its determinants among students: A structural equation modeling analysis
Source: PLoS One. 2022 Mar 21;17(3):e0263568. doi: 10.1371/journal.pone.0263568 (PMC8936445; doi:10.1371/journal.pone.0263568)
Supplement: Attachment — Submitted filename: Response to edditor and reviewers.docx [file pone.0263568.s006.docx]

**Dear Ali B. Mahmoud, Ph.D., editor and reviewers:**

Hereby, we resubmit the enclosed –revised- manuscript ID PONE-D-21-05732-[ EMID:15744fc9849b3e3d], which is entitled “Application of Health Belief Model for the Assessment of COVID-19 Preventive Behavior and its Determinants among Students: A Structural Equation Modeling Analysis” to your journal*.* We have read the comments carefully and we were able to implement all of them (**see the manuscript with track changes**). While reading the manuscript critically, we spotted English grammar errors, which we corrected. We hope that our revision will be felt like an improvement. Furthermore, we assessed our reference list for retracted papers and we didn’t cite any retracted article. Thank you!

**Response to reviewers and editors**

1) Suggestion to improve paper’s structure:

I suggest removing the first paragraph that introduces Covid-19. I find it to be too medically focused. There is no need to speculate on the origins of the virus (particularly since it is not established definitively) since it is irrelevant for the purpose of this paper. Alternatively, authors could replace medical wording with simple reference to Covid-19 pandemic. Everyone knows and understands what this is.

# I would suggest instead to incorporate some of the already established early research studies on negative effects of Covid-19 as applicable to either the same settings (such as educational environment), or the same demographics (such as kids). For example, the following paper summarizes the up-to-date research finding based on kids at-home eating habits during Covid:

# “The Janus-faced effects of COVID-19 perceptions on family healthy eating behavior: Parent’s negative experience as a mediator and gender as a moderator” by [Ali B. Mahmoud](https://onlinelibrary.wiley.com/action/doSearch?ContribAuthorStored=Mahmoud%2C+Ali+B), [Dieu Hack-Polay](https://onlinelibrary.wiley.com/action/doSearch?ContribAuthorStored=Hack-Polay%2C+Dieu), [Leonora Fuxman](https://onlinelibrary.wiley.com/action/doSearch?ContribAuthorStored=Fuxman%2C+Leonora), [Maria Nicoletti](https://onlinelibrary.wiley.com/action/doSearch?ContribAuthorStored=Nicoletti%2C+Maria) <https://onlinelibrary.wiley.com/doi/10.1111/sjop.12742>

Giving the reader the summary of findings about Covid related impacts within similar context would present a better introductory background to the current study.

***Response:*** *Correction has been made accordingly* ***(See line 67-82 on page 4 in the manuscript).***

2) I suggest deleting the entire section “Study Period and Setting”. The only immediately relevant information in this section are the dates of the study, which authors can easily incorporate into the next section.

***Response:*** *Correction has been made accordingly* ***(See line 156-165 at page 5 in the revised manuscript with track changes).***

This revision is an improved one. The paper needs detailed proof reading to reconcile English discrepancies. Please see my suggested modifications in the attached file.

**Response:** *Thank you for taking time to review our work for the language details. Proofreading and language review have been made throughout the manuscript* ***(See the revised manuscript with a track change).***

3) Here are some suggestions to correct language throughout the manuscript

Line 73: remove end of sentence punctuation (**See line 69-70 on page 4 in the manuscript**).

***Response:*** *Correction has been made accordingly*

Lines 75-78 (third paragraph): I don’t see the need to re-state mortality statistics as those do change rather quickly and are widely accessible online. This paper is about students in University setting and as such the focus of the introduction should be on the specifics of the environment under consideration.

Line 88-89: grammar correction : “more than 94% of students were following the recommended preventive health behaviors in Iran

***Response:*** *Correction has been made accordingly (****See line 93-94 on page 5 in the manuscript****).*

Line 106: correct punctuation – remove “; ”

**Response:** Correction has been made accordingly *(****See line 112 on page 6 in the manuscript****).*

Line 116: grammar correction – “The HBM is one of the various health behavior models that has been…”

***Response:*** *Correction has been made accordingly (****See line 122 on page 6 in the manuscript****).*

Lie 127: replace “study area” with academia.

***Response:*** *Correction has been made accordingly (****See line 134 on page 7 in the manuscript****).*

Line 136: replace “administration” with administered.

**Response:** Correction has been made accordingly

Line 147: replace “refers” with refer.

**Response:** Correction has been made accordingly *(****See line 145 on page 7 in the manuscript****).*

Line 151: and/or

**Response:** *Correction has been made accordingly (****See line 149 on page 7 in the manuscript****).*

Line 156: capitalize the title of the journal

***Response:*** *Correction has been made accordingly (****See line 153 on page 7 in the manuscript****).*

Line 162: replace punctuation “;” with :

***Response:*** *Correction has been made accordingly (****See line 159 on page 8 in the manuscript****).*

Line 163: coma needed ” … variable (0.43),”

***Response:*** *Correction has been made accordingly (****See line 160 on page 8 in the manuscript****).*

Line 167: “governmental schools, which resulted…”

**Response:** Correction has been made accordingly *(****See line 164 on page 8 in the manuscript****).*

Line 188: replace punctuation “;” with :

***Response:*** *Correction has been made accordingly (****See line 183 on page 9 in the manuscript****).*

Line 190: insert “is resource efficient”

**Response:** *Correction has been made accordingly (****See line 185 on page 9 in the manuscript****).*

Line 235: delete coma - “practice concerning handwashing, …”

***Response:*** *Correction has been made accordingly (****See line 235 on page 11 in the manuscript****).*

Line 244: add coma – “cleaning, coding…”

***Response:*** *Correction has been made accordingly (****See line 239 on page 11 in the manuscript****).*

Line 262: insert “ produce precise estimate with smaller…”

***Response:*** *Correction has been made accordingly (****See line 257 on page 12 in the manuscript****).*

Line 276: add coma – “perceived benefit, perceived barrier…”

***Response:*** *Correction has been made accordingly (****See line 271 on page 13 in the manuscript****).*

Lin 301: add reference for Declaration of Helsinki.

***Response:*** *The reference has been added ((****See line 296 on page 14 in the manuscript****).*

Line 312: replace “kept” with keep for grammatical consistency.

***Response:*** *Correction has been made accordingly (****See line 307 on page 16 in the manuscript****).*

Line 324: correct - “…scores were higher than the neutral score…”

**Response:** Correction has been made accordingly *(****See line 319 on page 18 in the manuscript****).*

Line 337: add coma and lower case – “perceived severity (r = 0.2, p < 0.05), perceived benefit…”

**Response:** Correction has been made accordingly *(****See line 332 on page 18 in the manuscript****).*

All statistical techniques, new and old, should have reference. For example, line 336 and 365

***Response:*** *Correction has been made accordingly (****See line 331 and 361 on page 18 and 361 in the manuscript, respectively****).*

Line 407-408: poor choice of wording “These results are results lower than the result reported by various previous studies…” Please rewrite this sentence

***Response:*** *Correction has been made accordingly (****See line 402-403 on page 24 in the manuscript****).*

Line 420: lower case “perceived barriers”

***Response:*** *Correction has been made accordingly (****See line 415 on page 25 in the manuscript****).*

Line 437: “…studies that were done using the health belief model…”

**Response:** Correction has been made accordingly *(****See line 432 on page 25 in the manuscript****).*

Line 440: use abbreviation HBM instead of health belief model.

**Response:** Correction has been made accordingly *(****See line 435 on page 25 in the manuscript****).*

Line 446: remove “is” or add our results “… the results are consistent…”

**Response:** Correction has been made accordingly *(****See line 441 on page 26 in the manuscript****).*

Line 449: replace punctuation “;” with:

**Response:** Correction has been made accordingly *(****See line 444 on page 26 in the manuscript****).*

Line 457: capitalize the name of the model to keep consistent “Health Belief Model”

**Response:** Correction has been made accordingly *(****See line 452 on page 26 in the manuscript****).*
